# When Does an Alien Become a Native Species? A Vulnerable Native Mammal Recognizes and Responds to Its Long-Term Alien Predator

**DOI:** 10.1371/journal.pone.0031804

**Published:** 2012-02-15

**Authors:** Alexandra J. R. Carthey, Peter B. Banks

**Affiliations:** Ecology and Evolution Research Centre, School of Biological, Earth and Environmental Sciences, University of New South Wales, Sydney, Australia; University of Pretoria, South Africa

## Abstract

The impact of alien predators on native prey populations is often attributed to prey naiveté towards a novel threat. Yet evolutionary theory predicts that alien predators cannot remain eternally novel; prey species must either become extinct or learn and adapt to the new threat. As local enemies lose their naiveté and coexistence becomes possible, an introduced species must eventually become ‘native’. But when exactly does an alien become a native species? The dingo (*Canis lupus dingo*) was introduced to Australia about 4000 years ago, yet its native status remains disputed. To determine whether a vulnerable native mammal (*Perameles nasuta*) recognizes the close relative of the dingo, the domestic dog (*Canis lupus familiaris*), we surveyed local residents to determine levels of bandicoot visitation to yards with and without resident dogs. Bandicoots in this area regularly emerge from bushland to forage in residential yards at night, leaving behind tell-tale deep, conical diggings in lawns and garden beds. These diggings were less likely to appear at all, and appeared less frequently and in smaller quantities in yards with dogs than in yards with either resident cats (*Felis catus*) or no pets. Most dogs were kept indoors at night, meaning that bandicoots were not simply chased out of the yards or killed before they could leave diggings, but rather they recognized the threat posed by dogs and avoided those yards. Native Australian mammals have had thousands of years experience with wild dingoes, which are very closely related to domestic dogs. Our study suggests that these bandicoots may no longer be naïve towards dogs. We argue that the logical criterion for determining native status of a long-term alien species must be once its native enemies are no longer naïve.

## Introduction

The devastating impact of alien predators on native wildlife within their new ranges [1], is often attributed to prey naiveté, or a failure to recognize and respond appropriately to a novel predation threat due to lack of experience [2], [3], [4]. Until recently, prey naiveté has been considered an all-or-nothing status, closely akin to island syndrome [2], [3]. Yet it is inarguably a more complex phenomenon, involving multiple levels through which native prey might progress towards predator-wariness [5], with each level closely tied to degrees of experience with a novel predator. It follows logically that alien predators cannot remain eternally novel; evolutionary theory predicts that prey species that do not become extinct must learn and adapt to new threats [6], [7], and there is recent evidence for this [e.g. 8]. According to some definitions [e.g. 9], [10], any species becomes irrevocably alien once it has been human-dispersed [11]. Yet the history of global biotic interchange, range expansion and natural dispersal shows that many native species were themselves once alien [12], and that adaptation and evolution will allow native communities to integrate many alien species, given sufficient time [6], [7], [13]. Naiveté of local species towards an alien threat cannot persist forever; therefore, alien status should not be considered immutable. In many cases, alien species will eventually become native. Despite an increasing focus on the effects of naiveté towards alien enemies soon after invasion [concentrated on], [ but not limited to predator]-[prey interactions; e.g. 2,4,14,15,16,17,18,19], the processes by which these novel ecological interactions between alien and native species develop and change in the medium to long term after establishment have received little attention [but see 13], [20]. As a result, one critical question remains unanswered: at what point after establishment and naturalization is an alien species sufficiently integrated into its new ecosystem to be considered native? We suggest that the logical, objective criterion that distinguishes this long-term change is the loss of ecological novelty, or in other words, the loss of naiveté of the alien species' native enemies in its new range.

Australia's fragile mammalian fauna have been heavily impacted by alien predators [21], with naiveté due to an isolated evolutionary history thought to underlie the rapid pace of extinctions and declines [1]. The dingo (*Canis lupus dingo*) was introduced to the continent approximately 4000 years ago, and may have caused extinctions and declines soon after its arrival and establishment [for example], [ the Tasmanian devil (*Sarcophilus harisii*), Tasmanian tiger (*Thylacinus cynocephalus*), and the Tasmanian native hen (*Gallinula mortierii*) disappeared from the mainland soon after the dingo's arrival, but persisted in Tasmania where the dingo never reached; 22,23]. Dingoes remain alternatively protected as a native species in some areas and persecuted as an introduced pest in others. It seems implausible that native prey species that have survived thousands of years (and hence generations) of dingo predation could continue to exhibit naiveté towards this introduced predator today. The feral cat (*Felis catus*, introduced ∼150 yrs ago) is implicated in current faunal declines [21], and the domesticated counterparts of both these predators (pet dogs, *C. lupus familiaris*, and cats, also *F. catus*) kill native wildlife at the bush-urban interface.

Native bandicoots (*Perameles nasuta*) are vulnerable, critical weight range [24] marsupials that regularly emerge from bush land to forage in residential yards adjacent to national parks in Sydney. Foraging bandicoots leave behind characteristically deep, conical holes in lawns and gardens, which reliably indicate their presence [25]. *C. familiaris* and *C. dingo* are so closely related that they readily interbreed in the wild, and distinguishing between pure dingoes and wild dogs has become a conservation concern in Australia [26]. If long-term experience with an alien predator reduces the level of naiveté shown by native prey species, we would predict that bandicoots' far longer coexistence history with dingoes compared to feral cats should enable them to recognise the predation risk posed by domestic dogs but not cats. We therefore expected to see fewer and less frequent signs of bandicoot activity in yards with resident dogs than in yards with cats or no pets. To test this prediction, we assessed hundreds of replicates of predator presence or absence by surveying local residents to determine the level of visitation of bandicoots to their back yards. We compared responses from households without pets, to those with dogs or cats, whilst controlling for other variables such as yard size and accessibility. Our survey and analysis were designed to test whether Australian bandicoots remain naïve to dogs despite thousands of years experience with dingoes.

## Materials and Methods

### Ethics statement

All work was conducted with the approval of the University of New South Wales Human Research Ethics Advisory Panel (UNSW HREAP Approval Number 1205). Written consent was not obtained, because participants were free to choose whether to complete and return the survey or not; hence completion of the survey was considered to indicate consent. This form of consent was approved by the UNSW HREAP.

We delivered two thousand surveys to properties adjacent to national parks in Sydney, where bandicoot foraging in lawns is regularly reported. The distinctively-shaped holes left by foraging bandicoots are well-recognized by residents, but survey invitations contained example images of bandicoots and their diggings, as well as those of local non-target animals (black rats – *Rattus rattus*, possums – *Trichosurus vulpecula*, and rabbits – *Oryctolagus cuniculus*). The survey invitation requested responses from all residents, including those who had not observed bandicoots or their diggings. Questions were multiple choice and allowed comments. Residents were requested to consider their backyards only, to standardize responses, and because that is where pets are generally kept. The survey requested that residents report on sightings of bandicoots, the presence of their diggings, the frequency with which fresh diggings typically appeared in their back yards, and the quantity of fresh diggings that would typically appear. Residents were also asked to report the number of cats and dogs that they owned, the size of their dogs, and how frequently their pets were kept inside during the day and at night. We controlled for other yard characteristics that might affect the occurrence of bandicoots and their diggings by including questions about yard size, perceived accessibility to a bandicoot, watering frequency (as bandicoots prefer to dig in moist soil), whether the yard was mostly garden or paved, and whether pet food was left outside overnight (in case it attracted bandicoots). 227 responses were received. After removing bandicoot-inaccessible and paved yards from the analysis, 197 yards remained. Of these, 97 had no resident pets, 52 had dogs, and 36 had cats (12 had both, but were excluded due to small sample size). We used exact chi-squared tests of independence [see 27, and File S1] to compare bandicoots' use of yards with dogs or cats to yards with no pets, and to test whether keeping dogs and cats inside, or any of the yard characteristics were associated with signs of bandicoot activity. Adjusted standardized residuals (ASR's) were calculated for contingency tables to determine where differences lay; ASR's>|2| indicate a directional lack of fit of the null hypothesis (that factors are independent) in that cell [28]. All statistics were performed using the exact tests module in PASW Statistics v18, IBM Statistics, 2010.

## Results

**Figure 1:**
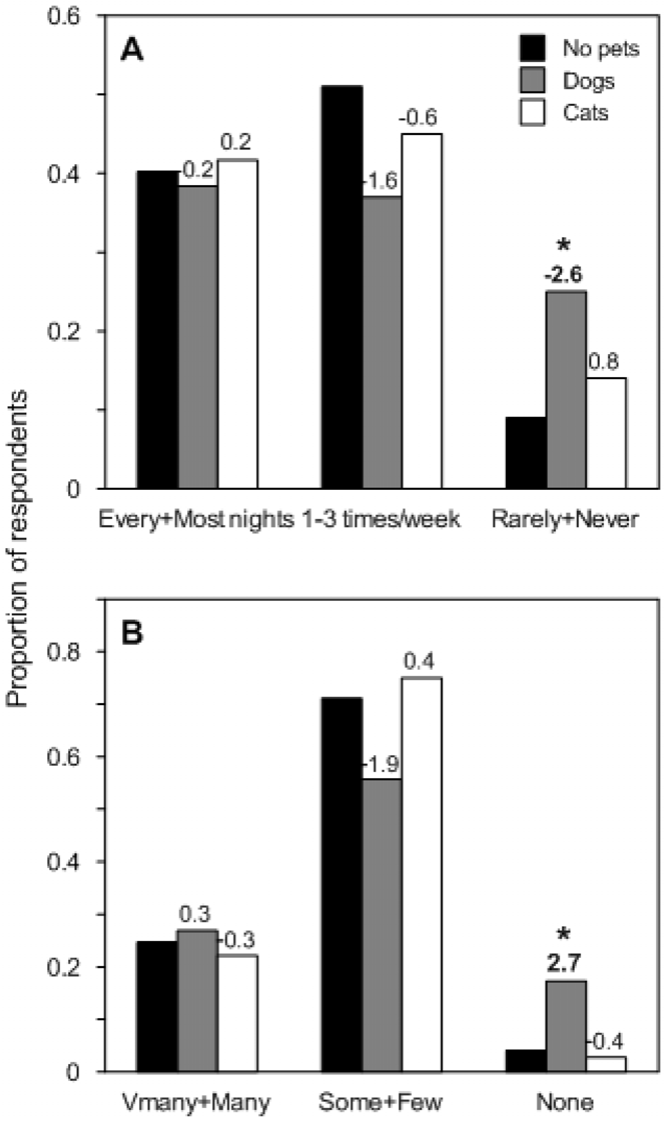
Typical frequency (A) and quantity (B) of diggings appearing in yards with each pet type. Dog owners were more likely to report rarely or never seeing fresh diggings (A), and seeing no new diggings (B). Data are proportions of survey respondents choosing each answer. Numbers above the bars are adjusted standardized residuals from the contingency analysis of each question for dogs versus no pets and cats versus no pets. Residuals greater than two indicate a lack of fit of the null model in that cell (denoted by asterisks). Negative residuals indicate a smaller proportion choosing that answer, and positive residuals indicate a greater proportion choosing that answer than expected if factors were independent.

The presence of dogs was associated with fewer signs of bandicoots: fewer yards with dogs had diggings at all (ASR = −2.4, *p* = 0.02), and these yards had less frequent (Fig. 1a, *p* = 0.03), and lower quantities of fresh diggings (Fig. 1b, *p* = 0.02) than did yards without pets. The presence of cats was not associated with any of these measures (Fig. 1; all ASR's<|2|; all *p*>0.05). Having multiple dogs in a yard made no difference compared to a single dog, nor did dog size have any effect (Table 1). Neither keeping pets indoors nor leaving pet food outside at night made any difference to the reported appearances of bandicoot diggings in yards (Table 1), but respondents with larger (>50 m^2^) yards reported greater quantities of diggings (ASR = 2.2; *p* = 0.03), frequent watering was associated with fresh diggings appearing most or every night (ASR = 2.8; *p* = 0.02), and all-lawn yards were more likely to have diggings appear than half-paved yards (ASR = 2.2; *p* = 0.04). However, these control variables were unrelated to the type of pet owned (Table 2). Bandicoot sightings were unrelated to the presence of either type of pet (dogs: *p* = 0.58; cats: *p* = 0.38).

**Table 1:** Bandicoot activity was not affected by size, number of pets, or whether pets or food were outside overnight.

Variable		Exact *p*	
	*Presence of diggings*	*Frequency of diggings*	*Quantity of diggings*
*Number of dogs*	0.67	0.48	0.73
*Dog size category*	0.26	0.14	0.73
*Dogs inside at night*	0.84	0.12	0.69
*Cats inside at night*	1.00	0.19	0.33
*Pet food outside overnight*	0.63	1.00	0.38

Results of the χ2 tests of independence for each variable vs. each of three measures of bandicoot activity in yards. Values are the exact probability (significance evaluated at α = 0.05) that each contingency table would occur if that particular combination of variables were independent.

**Table 2:** Yard size, paving and watering frequency were not related to the type of pet owned.

Variable	Exact *p*
	*Pet type owned*
*Yard size*	0.33
*Frequency of watering*	0.79
*Paved or all-lawn yard*	0.28

Results of the χ2 tests of independence for control variables vs. type of pet owned. Values are the exact probability (significance evaluated at α = 0.05) that each contingency table would occur if that particular combination of variables were independent.

## Discussion

Our results suggest that native bandicoots recognize the threat posed by dogs but not cats when choosing where to forage. More than twice the proportion of respondents who owned dogs in our study reported seeing fresh diggings rarely or never (Fig. 1A), or not seeing bandicoot diggings at all (Fig. 1B), compared to respondents owning either cats or no pets. Two respondents indicated that their dog had killed a bandicoot within the past 6 months, and bandicoots are found in dingo stomachs [22], confirming that the threat to bandicoots from dogs is real. Whilst no killings by cats were reported (and this information was not specifically requested), domestic cat predation of bandicoots is well documented, including from the Sydney area [29] and feral cat predation lead to the demise of the closely related eastern barred bandicoot (*Perameles gunnnii*) in suburban Melbourne [30].

Most respondents in our survey (73.1% dogs; 80.6% cats) allowed their pets outside at night once per week or less, and keeping pets outside more often was not associated with fewer signs of bandicoots (Table 1). Nocturnal bandicoots would rarely encounter these house-bound pets, suggesting that reduced signs of activity are not simply due to bandicoots being chased away or killed by resident dogs; instead they appear to recognize and avoid the danger represented by resident dogs.

These findings support our hypothesis that ∼4000 years of experience with the dingo have been sufficient for native wildlife to recognize and respond to the predation risk of dogs. That is, to these bandicoots, dogs may no longer be a *novel* predation threat. Research shows that Australian wildlife are capable of rapid learning and adaptation in response to novel threats [31], [32]. More recently, it has been suggested that dingoes suppress mesopredators such as cats and foxes, with a net benefit outcome for the biodiversity of smaller native mammals in particular [33], [34]. We speculate that wariness of native prey towards dingoes, combined with continuing naiveté towards the more recent invaders, cats and foxes, could potentially amplify the effects of these interactions. Davis *et al* [13] argue that such potentially beneficial interactions with native species should be more important than a species' origin for management and conservation decisions.

Just how much exposure to local enemies is necessary for an alien species to lose its novelty? The apparent lack of response to cats by bandicoots in our study suggests that for this particular pairing, hundreds of years of coexistence may not be enough. However, cats may pose different risks to dogs as they are more mobile and range past the boundaries of a particular yard, although their activity will be concentrated in that area. Experimental field manipulation of exposure to predators could examine this question.

Naiveté towards novel threats occurs not only between predators and prey, but in any type of antagonistic ecological interaction resulting from the introduction of a species into a new range, such as competitive, host-parasite, and plant-herbivore interactions [e.g. 19]. More generally, every novel enemy-enemy pairing in a new range will begin at an initial level of naiveté *sensu* [5], possibly determined by the similarity of the new species to native ones, either functionally [e.g. ‘predator archetypes’ 2], in their appearance [e.g. predators may smell similar due to meat metabolites; 35], or through phylogenetic relationships [36]. Over time, local species will either go extinct or learn and adapt to the introduced threat [6], [7]. Arbitrary cut-off dates delineating native from alien species are not scientifically founded. We suggest that the only objective criterion for deciding whether an introduced species has sufficiently integrated to be considered native must ultimately be the loss of novelty - that is, when native species recognize and respond effectively to the introduced enemy. The preliminary results presented here indicate that this process may have begun for the dingo in Australia.

## Supporting Information

File S1 **Details of contingency table analysis and category pooling**.
(DOC)
