# Supplementary material for: When Does an Alien Become a Native Species? A Vulnerable Native Mammal Recognizes and Responds to Its Long-Term Alien Predator
Source: PLoS One. 2012 Feb 15;7(2):e31804. doi: 10.1371/journal.pone.0031804 (PMC3280208; doi:10.1371/journal.pone.0031804)
Supplement: File S1 — Details of contingency table analysis and category pooling. (DOC) [file pone.0031804.s001.doc]

**Supporting Information. Details of contingency table analysis and category pooling**

Despite the large overall sample size, some cells were sparsely populated in many contingency tables due to the nature of the questions. To overcome this issue, some categories were pooled, and exact probabilities for chi squared tests of independence were calculated, hence neither DF nor the χ^2^ test statistic are reported. For the frequency of diggings question, the response categories were pooled into ‘Never or rarely’, then ‘Between once and three times per week’, and then ‘Most nights or every night’. For the quantity of diggings, the response categories ‘Few’ and ‘Some’ were pooled, as were the categories ‘Many’ and ‘Very many’. Dog size classes were grouped into ‘Very small to medium’, and then ‘Large and very large’. Yard sizes were grouped into ‘Small - medium’, and then ‘Large and very large’ categories. Responses for yard watering frequency were pooled into ‘Daily’ and ‘Several times per week’, and then those who watered ‘Once per week or less often’. Responses for the question about pet food being left outside at night were pooled into two groups: those who replied ‘Yes’ (including those who left pet food outside often, and those who left it outside overnight occasionally) and those who replied ‘No’ (they never left pet food outside overnight).
